# Interleukin-2 in Renal Cell Carcinoma: A Has-Been or a Still-Viable Option?

**DOI:** 10.15586/jkcvhl.2014.18

**Published:** 2014-11-23

**Authors:** Asim Amin, Richard L White

**Affiliations:** 1 Divisions of Immunotherapy and Medical Oncology, Levine Cancer Institute, Carolinas Medical Center, Charlotte, North Carolina, USA; 2 Divisions of Surgical Oncology and Immunotherapy, Levine Cancer Institute, Carolinas Medical Center, Charlotte, North Carolina, USA.

## Abstract

Modulation of the immune response plays an important role in the natural history of renal cell carcinoma. Spontaneous regression of metastases has been well documented in a small percentage of patients after they undergo de-bulking nephrectomy without any additional systemic intervention. The only logical explanation for these observations is “resetting” of the balance between tumor and the host immune system that, having been overwhelmed by the tumor burden, is able to function better after tumor de-bulking. Attempts to modulate the activity of the immune system “on demand” have included the use of vaccines, cytokines/lymphokines, adoptive cell transfer, monoclonal antibodies and most recently manipulation of immune checkpoint inhibitors. Here we review the data for infusional interleukin-2 in the management of advanced renal cell carcinoma and its role in current clinical practice.

## Introduction

Spontaneous regression of metastatic renal cell carcinoma has been reported in a small percentage of patients after de-bulking nephrectomy without any additional systemic intervention (^1-3^). This is thought to be the result of “resetting” of the host immune system that had been overwhelmed by the tumor burden. Hence, immunotherapy has been the mainstay of treatment for advanced renal cell carcinoma until the introduction of targeted therapies. Interleukin 2 (IL-2) was approved by the USFDA in 1992 for the treatment of advanced renal cell carcinoma.

## Interleukin-2

Demonstration that T lymphocytes could be grown in vitro, only in the presence of conditioned medium from phytohemagglutinin (PHA)-stimulated human blood lymphocytes (4), led to the discovery of a T cell growth factor subsequently designated IL-2 (5,6,7). T lymphocytes grown in IL-2 containing culture were shown to have the ability to kill tumor cells in vitro (8). IL-2 activated human peripheral blood lymphocytes showed lysis of natural killer-resistant fresh solid tumor cells - these were termed LAK cells (9). IL-2 was deemed to be necessary and sufficient for T cell growth and activation. In vivo animal studies demonstrated that adoptive immunotherapy with transfer of syngeneic LAK cells generated in vitro, using IL-2, could eliminate natural, killer-resistant, established pulmonary melanoma and sarcoma metastases (10, 11). IL-2 was shown to stimulate in vivo proliferation of adoptively transferred LAK cells (12), and systemic administration of high-dose IL-2 without adoptive T cell transfer was shown to cause regression of established pulmonary metastases and subcutaneous tumors, proving that LAK cells could be generated in vivo (13). The cDNA coding for IL-2 was cloned and was shown to consist of 153 amino acids with a molecular weight of 15,420 daltons (14). Availability of IL-2 in large quantities made clinical trials possible. Rosenberg et al. reported their experience in 25 treatment-resistant patients with advanced cancer, who were treated with a combination of LAK cells and interleukin-2. These included patients with malignant melanoma, colorectal cancer, sarcoma, renal cell carcinoma, non-small cell lung cancer and esophageal cancer. Eleven out of 25 patients had marked tumor regression; one patient with metastatic melanoma had a complete remission while 10 partial responses were observed, thus establishing proof of the principle that manipulation of the immune system using high-dose IL-2 could be performed safely and would induce significant clinically relevant responses (15).

The discovery and availability of IL-2 for clinical use was pivotal in bringing an immunotherapeutic modality to the forefront (16). Given that immune-mediated regression had been observed in patients with renal cell carcinoma and the fact that renal cell carcinoma does not respond to chemotherapy, the earliest clinical investigations with IL-2, carried out at the NIH Surgery Branch, included renal cell carcinoma. A progress report on the treatment of 157 patients with advanced cancer, using LAK cells and IL-2 or high-dose IL-2 alone, included 36 patients with renal cell carcinoma. An impressive 33% response rate was observed: 4/36 had a complete response and 8/36 had a partial response (≥ 50% decrease in sum of the products of the perpendicular diameters of all lesions). An additional 7/36 patients experienced a minor response (25 to 49% decrease in sum of the products). Most of the patients who had a complete response had lung metastases (17).

## High-dose IL-2 in RCC

Further work at the NCI Surgery Branch reported their experience in 283 patients with metastatic melanoma or metastatic renal cell cancer treated from September 1985 through December 1992 with high-dose bolus IL-2– this series included 149 patients with renal cell carcinoma. Patients received IL-2 at the dose of 720,000 international units per kilogram intravenously every 8 hours for a maximum of 15 doses per cycle: 2 cycles constituted a course of therapy. Patients who showed response or stable disease after the first course went on to receive additional therapy. An overall response of 20% (CR+PR) was observed in patients with renal cell carcinoma, 7% (n=10) achieved complete response, and 13% (n=20) had a partial response. With the exception of one complete responder who had liver metastases, all others had lung metastases or involvement of lymph nodes. The responses were noted to be durable and ongoing at up to 76 months in the patients with a complete response, and 69 months in those with a partial response at the time of publication. There were 3 (1.1%) treatment-related deaths; 2 due to myocardial infarction and one as a result of sepsis (18).

Some of the selected trials using high-dose IL-2 are summarised in Table 1. A large additional series published by Fyfe et al. reported the outcomes of 255 patients with advanced renal cell carcinoma treated with high-dose bolus IL-2 enrolled in 7 phase II studies at 21 institutions. Two of these studies used the dose of 720,000 international units per kilogram while the other 5 used the dose of 600,000 international units per kilogram administered every 8 hours over 15 minutes for a total of 14 doses per cycle. Responses were observed in 37/255 patients (15%); 17 (7%) patients achieved a complete response and 20 patients (8%) a partial response. The updated median duration of response for all patients was 54 months. The median duration for those with complete response had not been reached at the time of reporting: it was at least 18 months with a range of 7 to greater than 131 months. The median duration of response for the patients who had a partial response was 20 months with a range of 3 to greater than 126 months. Eleven (4%) out of 255 patients died of treatment-related toxicity (6 patients had a myocardial infarction or respiratory failure, 3 had gastrointestinal toxicity, one had sepsis and one died at home of unknown causes) (19, 20).

**Table 1.** High dose interleukin-2 in advanced RCC

Study	N	Dose	ORR	Response Duration (Months)	Ref.
**7 phase II** **studies**	255	600,000-720,000 IU/kg	ORR*= 15% CR= 7% PR= 8%	54 (3 to 131+) 80+ (7 to 131+) 20 (3 to 126+)	(19,20)
**NCI experience** **Single** **Institution**	259	720,000IU/kg	20% CR=9% PR =11%	21 CR median survival not reached – 221+	(21)
**Phase II “SELECT” study**	120	600,000IU/kg	29%	20 responders (4 to 35+)	(22)

* CR, complete response; ORR, overall response rate; PR, partial response

Klapper et al. reported the expanded NCI Surgery Branch experience for 259 patients with advanced renal cell carcinoma who were treated between 1986 and 2006 with high-dose IL-2 at the dose of 720,000 units per kilogram IV bolus every 8 hours up to a total of 15 doses per cycle (later reduced to a maximum of 12 doses). Response was evaluated using the WHO criteria initially and RECIST criteria since 1998. An overall objective response rate of 20% was observed (consistent with previous reports); 9% (23/259) of patients achieved a complete response and 12 % (30/259) of patients had a partial response. Median survival for the partial responders was 39.1 months and had not been reached for the complete responders at the time of analysis. There were 2 treatment-related deaths in the earlier part of the study (21).

In an effort to optimize IL-2 therapy, the renal cell “SELECT” trial was designed to assess if the patient population could be selectively enriched based on carbonic anhydrase IX overexpression within the tumor besides other factors. Of the 120 patients treated in this study, 96% had clear cell histology and 99% had nephrectomy. Patients received high-dose IL-2 at the dose of 600,000 international units per kilogram every 8 hours. The overall response rate was 29% (35/120): the response rate for clear cell histology was 30% (35/115). A complete response was observed in 7 patients, while 28 patients had a partial response. Although no correlation was noted with carbonic anhydrase IX expression, clear cell histology appeared to be important given the higher than historically observed response rates (22).

## High dose vs/or low dose IL-2

Despite the durable responses elicited by high-dose IL-2 that may translate into “cure” for some, its use is restricted to selected individuals with good organ function and performance status. Wider applicability is limited due to significant toxicity in the form of a brisk capillary leak syndrome that can result in multi-organ system compromise, albeit rapidly reversible but requiring intense monitoring and trained personnel in place. Efforts have therefore been made to study the efficacy of IL-2 at lower doses in the hope that treatment might be safer and easier to administer to a broader patient base. Some of the selected trials using low-dose IL-2 and comparing them to high-dose regimens are discussed (Table 2).

**Table 2.** High dose interleukin-2 vs/or low dose interleukin-2 in advanced RCC

Study	N	Dose	ORR	Response Duration (months)	Ref.
**Randomized** CIV* IL2 **SC IFN α** **CIV IL2 + SC IFN α**	138 147 140	18x106 IU/m2/d CIVx5d 18x106 IU/d sc 3x/w 18x106 IU/m2/d CIVx5d + IFN 6x106 sc 3x/w	6.5% 7.5% 18.6%	Median OS 12 Median OS 13 Median OS 17	(23)
**Randomized** **High dose bolus IL2** **Low dose bolus IL2**	**155** 149	**720,000 IU/kg** 72,000IU/kg	**21%** (CR=11, PR=22) 13% (CR=6, PR=13)	8/11 ongoing CR at 9.3 yrs 3/6 ongoing CR at 10.1 yrs	(24)
**Randomized** **High dose bolus IL2** **SC Low dose IL2** **+** **IFN**	**95** 91	**600,000IU/kg** IL2 5x106 IU/m2 q8hr d1 Then 5x106IU/m2 qd x4d + IFN 5x106IU/m2 3x/w	**23.2% (CR=8)** 9.9% (CR=3)	14 (10/22 free of progression at 3 years) 7 (3 pts free of progression at 3 years	(25)
**Randomized** **Medroxyprogesterone** **SC IFN** **SC Low dose IL2** **SC IFN + IL2**	123 122 125 122	200 mg orally every day 9x106 IU 3x/w 9x106 IU bid x 5d week 1 9x106 IU bid x 2d then 9x106 IU q d x 3d, w2-4 IL2 and IFN schedule as above; IFN dose 6x106IU	2.5% 4.4% 4.1% 10.9%	Median OS 14.9 Median OS 15.2 Median OS 15.3 Median OS 16.8	(26)

* CIV, intravenous; CR, complete response; ORR, overall response rate; OS, overall survival; PR, partial response; Sc, subcutaneous

In that vein, Negrier et al. reported 425 patients with metastatic renal cell carcinoma, randomized to receive IL-2 as a continuous intravenous infusion or interferon alpha 2a or the combination of both (23). In the IL-2 monotherapy arm induction phase, drug was administered at the dose of 18 million international units per square meter of body surface area per day for 5 days repeated twice with a 6-day break. In the maintenance phase, the same dose was repeated every 3 weeks. In the interferon monotherapy arm, drug was administered at the dose of 18 million international units subcutaneously 3 times a week for 10 weeks as induction and 13 additional weeks as maintenance. In the combination arm, IL-2 was administered at full dose (as above) but interferon alpha 2a was administered at the dose of 6 million international units 3 times a week during IL-2 dosing. The response rates were 6.5% for low dose IL-2 monotherapy, 7.5% for interferon alpha 2a monotherapy and 18.6% for the combination. There was no significant difference in overall survival, and toxicity was greater in the IL-2 containing arms.

The Surgery Branch at the NCI conducted a 3 arm randomized study comparing high-dose IL-2 to two different low-dose IL-2 regimens (24). A total of 156 patients were assigned to the high-dose arm and received IL-2 at the dose of 720,000 international units per kilogram IV bolus every 8 hours.150 patients received low-dose IL-2 at the dose of 72,000 international units per kilograms IV bolus every 8 hours, while 94 patients in the third arm received daily subcutaneous low-dose IL-2. The response rate in the high-dose arm was 21%, 13% in the low-dose intravenous arm, and 10% in the daily subcutaneous. There were no treatment-related deaths. Toxicities, especially hypotension, were less frequent in the low-dose arms. Durable responses were noted in all arms: 8/11 patients who achieved a complete response in the high-dose arm were noted to have an ongoing response at 9.3 years. Three of the 6 patients who achieved complete response in the low-dose intravenous arm were noted to have ongoing response at 10.1 years and one complete response in the subcutaneous low-dose arm had an ongoing response past 78 months at the time of analysis.

The Cytokine Working Group further conducted a randomized phase III study to compare high-dose IL-2 with the combination of low-dose subcutaneous - IL-2 and interferon (25). In this study 91 patients received outpatient subcutaneous IL-2 at a loading dose of 5 million units per body meter squared every 8 hours for 3 doses on day 1 followed by 5 million units per meter squared on days 2, 3, 4 and 5 during the first week and then 5 million units per body meter squared for 5 days every week for 3 weeks. In addition these patients received interferon at the dose of 5 million units per meter squared thrice weekly for 4 weeks. A total of 95 patients were treated on the high-dose intravenous IL-2 arm and received 600,000 units per kilogram IV bolus every 8 hours for a maximum of 14 doses. An overall response of 23.2% (22/95) was observed in the high-dose intravenous IL-2 arm compared to 9.9% (9/91) in the low-dose subcutaneous IL-2 plus interferon arm. Eight patients achieved a complete response in the high-dose intravenous IL-2 arm; this response was durable in 7/8 patients at 3 years. Three of 9 responding patients achieved a complete response in the low-dose subcutaneous IL-2 plus interferon arm, but all of them had recurrence of disease within 3 years.

The French immunotherapy intergroup conducted a randomized trial to compare low dose subcutaneous IL-2, interferon alpha 2a, the combination of the 2 cytokines and medroxyprogesterone in 492 patients with metastatic renal cell carcinoma of intermediate prognosis (26). Intermediate prognosis was defined by requiring a Karnofsky score of ≥80% if metastases in more than one organ; Karnofsky score of 80% if metastases in one organ; normal blood, liver and renal function. Patients with prior treatment, evidence of brain metastasis, uncontrolled cardiac issues, active infections or who were on corticosteroids were excluded. Subcutaneous IL-2 was administered at the dose of 9 million international units every day to 125 patients. 122 patients received interferon alpha 2a at the dose of 9 million international units 3 times a week. 122 patients received both interferon and IL-2 at the above doses, and 123 patients received medroxyprogesterone acetate at the dose of 200 mg every day. The response rate was 3.3% in the IL-2 arm, 3.5% in the interferon arm, 10.9% in the cytokine combination arm, and 1.7% in the medroxyprogesterone arm. No differences were noted in the progression-free survival or overall survival amongst all the arms.

## High Dose IL-2 – Contemporary Experience: “PROCLAIM”

Based on the relatively poor results associated with low-dose IL-2 high-dose IL-2 has generally come to be accepted as the standard of care for properly selected patients. Since the early trials that led to the approval of high dose IL-2, centers of excellence have developed treatment schema that can greatly reduce toxicities. Mitigation strategies and guidelines to safely administer high-dose interleukin-2 have since been developed and used effectively (27). Data from contemporary single-institution/group series show that high-dose IL-2 can be administered safely at centers with adequate experience and careful selection of patients. At the Levine Cancer Institute, we have treated 104 patients with advanced renal cell carcinoma with high-dose IL-2. The response rate has been 26% including 8 complete and 19 partial responses. Five of the 8 complete responders have an ongoing response at 122 months. There has been no treatment-related mortality (28). Roswell Park Cancer Institute reported their experience for 91 patients who were treated with high-dose IL-2. The overall response rate was 16% with no treatment-related mortality (29). Payne et al. reported the experience from the Earle A Chiles Research Institute for 186 patients with advanced renal cell cancer treated with high-dose IL-2. The overall response rate was 24% with treatment-related mortality of less than 1% (30). The numbers treated at individual centers are however modest, making these non-systematic reviews difficult to interpret. In order to capture the contemporary experience more effectively, a high-dose IL-2 registry was established incorporating 35 centers in the USA (31). PROCLAIM (Proleukin® Observational Registry to Evaluate the Treatment Patterns and Clinical Response in Malignancy) is designed to create a robust observational database of real world contemporary high-dose IL-2 experience.

The goals of the PROCLAIM registry are to: (i), provide information regarding IL-2 and its prospective use; (ii), compare the difference in administration approaches for their respective effect on outcomes; (iii), validate efficacy of high-dose IL-2 on response and survival in the treatment of malignant diseases; (iv), identify patients and site-specific prognostic factors and (v), study and potentially guide the emergence of new therapeutic options in the immunological armamentarium.

The registry started enrolling the prospective cohort in September 2011; data for a retrospective cohort was collected between January 2007 and February 2012.

Despite the compelling high dose IL-2 data, many oncologists now employ targeted therapy with suppression of the VEGF or mTOR pathways as initial therapy in patients with advanced renal cell carcinoma. Retrospective reports with small numbers of patients treated with high-dose IL-2 after treatment with targeted therapy suggest higher than expected cardiovascular toxicity (some quite profound including cerebral vasculitis) if patients have previously received VEGF-targeted therapy (32). The PROCLAIM registry was queried to address the question of optimal sequencing of IL-2 therapy with targeted therapy. Observations from the retrospective data cohort presented by Morse et al. (33) showed the median overall survival for patients (n=82) who received high-dose IL-2 as initial systemic treatment to be 61.8 months compared to 48 months for those (n=15) who had received targeted therapy prior to high-dose (IL-2). While not conclusive, this observation suggests superior results in patients treated with high dose IL2 as first line therapy.

## Discussion

Immunotherapy with high-dose IL-2 had been the mainstay of systemic treatment for advanced renal cell carcinoma until the development of agents inhibiting the VEGF and mTOR pathways. VEGF TKIs (sunitinib, pazopanib, axitinib, sorafenib), mTOR inhibitors (temsirolimus, everolimus) and the combination of bevacizumab plus interferon have been approved for treatment of patients with metastatic renal cell carcinoma by the US FDA (34). Availability of these agents has had an impact on the natural history of the disease with prolongation of median survival to 22 months (35). Durable long-term responses observed with immunotherapy have, however, rarely been reported with these agents.

Given that treatment with high-dose IL-2 is not applicable for many patients with advanced renal cell carcinoma, new immunotherapeutic approaches are being investigated. Nivolumab is a fully human antibody that binds to PD-1, an immune inhibitory checkpoint expressed on the surface of T cells. Binding of the anti-PD-1 antibody to PD-1 blocks the interaction with its ligands PD-L1 and PD-L2 and prevents the T effector cell from switching off (36, 37). A phase II trial of nivolumab monotherapy in patients with metastatic enal cell carcinoma showed an overall response rate of 20% (using RECIST criteria v1.1) in heavily pretreated patients who had received up to 3 prior lines of treatment. The median overall survival for patients having received more than 2 lines of prior treatment was 18.7 months and was not reached in patients who had received one prior line of therapy. Almost one third of the responding patients showed ongoing responses beyond 24 months. Patients’ overall tolerance was reported to be good with grade 3-4 treatment-related ‘select’ events (defined as having potential immunologic etiology that require frequent monitoring and/or unique intervention) observed in less than 5% of the patients (38).

Hammers et al. presented data from a phase I study with the combination of nivolumab plus ipilimumab in patients with metastatic renal cell carcinoma. Ipilimumab is a fully humanized IgG1 antibody that binds to the immune inhibitory checkpoint CTLA-4. Binding of anti-CTLA-4 antibody to CTLA-4 expressed on the surface of activated T cells prevents the T cell from switching off (39). Ipilimumab has been approved for treatment of patients’ metastatic melanoma (34). Almost 80% of the patients in the study had received at least one prior line of treatment prior to receiving the combination. The overall response rate for nivolumab at 3mg/kg and ipilimumab 1 mg/kg was 43%: 78% of the responders had ongoing responses at the time of analysis (24 weeks). The combination of the two drugs was tolerated well with treatment-related ‘select’ adverse events (defined as above) observed in less than 5% of the patients (40). Amin et al. reported data for the phase I study with the combination of nivolumab with either sunitinib or pazopanib in patients with metastatic renal cell carcinoma. The overall response rate was 52% and 45% in the sunitinib and pazopanib combination arms respectively. While there were no treatment-related deaths, 82% in the sunitinib arm and 70% in the pazopanib arm experienced grade 3-4 treatment-related toxicities (41). These immunotherapeutic approaches hold great promise but mature data addressing the durability of these responses is awaited.

## Conclusion

Where does standard high-dose interleukin-2 fit in clinical practice with currently available options? The evidence demonstrates that properly selected patients with metastatic kidney cancer treated with IL-2 can be cured of their disease. Administration of high-dose IL-2 requires admission to hospital for one week at a time with close monitoring by a dedicated team. During the course of treatment patients can have a flu-like syndrome and experience substantial grade 3-4 toxicity secondary to capillary leak syndrome manifesting as hypotension, oliguria, generalized edema, tachy-arrhythmias and hypoxemia. These toxicities, however, occur in a monitored setting and are readily reversible. As experience with high-dose IL-2 and management of its toxicities has matured, the incidence of treatment-related irreversible high-grade toxicity including death has been minimized, as evidenced by data being collected from experienced centers in the PROCLAIM registry. Mortality rates in experienced centers are less than 1%. While the oral route of administration, non-life-threatening toxicities and broader applicability to most patients makes targeted therapies extremely desirable, daily dosing with ongoing side effects can significantly impact quality of life that needs to be balanced against the unlikely chance of a durable response. Additionally the side effects that may include diarrhea, stomatitis, fatigue, cardiac toxicity, increased incidence of hemorrhagic and embolic phenomena, hand-foot skin syndrome, elevated triglyceride levels, hyperglycemia and noninfectious pneumonitis occur in the non-monitored environment. Appropriate intervention for control of these symptoms is, therefore, largely dependent on patient reporting and outpatient office response mechanisms.

Emerging data for new immunotherapeutic modalities is extremely exciting and holds the promise of durable responses using agents administered in an outpatient setting, safely offered to a broad patient population. Given the established durability of responses with high-dose IL-2 and the ability to administer it safely at centers with experience, our institutional approach, supported by the NCCN guidelines (42), currently includes high-dose IL-2 as first-line, potentially curative therapy.
